# Preference-adaptive randomization in comparative effectiveness studies

**DOI:** 10.1186/s13063-015-0592-6

**Published:** 2015-03-18

**Authors:** Benjamin French, Dylan S Small, Julie Novak, Kathryn A Saulsgiver, Michael O Harhay, David A Asch, Kevin G Volpp, Scott D Halpern

**Affiliations:** Department of Biostatistics and Epidemiology, University of Pennsylvania, 423 Guardian Drive, Philadelphia, 19104 PA USA; Department of Statistics, University of Pennsylvania, 3730 Walnut Street, Philadelphia, 19104 PA USA; Department of Medicine, University of Pennsylvania, 3400 Spruce Street, Philadelphia, 19104 PA USA; Department of Health Care Management, University of Pennsylvania, 3641 Locust Walk, Philadelphia, 19104 PA USA; Department of Medical Ethics and Health Policy, University of Pennsylvania, 3401 Market Street, Philadelphia, 19104 PA USA; Center for Health Equity Research and Promotion, Philadelphia Veterans Affairs Medical Center, 3900 Woodland Avenue, Philadelphia, 19104 PA USA

**Keywords:** Adaptive design, Adherence, Comparative effectiveness research, Efficacy, Instrumental variables

## Abstract

**Background:**

Determination of comparative effectiveness in a randomized controlled trial requires consideration of an intervention’s comparative uptake (or acceptance) among randomized participants and the intervention’s comparative efficacy among participants who use their assigned intervention. If acceptance differs across interventions, then simple randomization of participants can result in post-randomization losses that introduce bias and limit statistical power.

**Methods:**

We develop a novel preference-adaptive randomization procedure in which the allocation probabilities are updated based on the inverse of the relative acceptance rates among randomized participants in each arm. In simulation studies, we determine the optimal frequency with which to update the allocation probabilities based on the number of participants randomized. We illustrate the development and application of preference-adaptive randomization using a randomized controlled trial comparing the effectiveness of different financial incentive structures on prolonged smoking cessation.

**Results:**

Simulation studies indicated that preference-adaptive randomization performed best with frequent updating, accommodated differences in acceptance across arms, and performed well even if the initial values for the allocation probabilities were not equal to their true values. Updating the allocation probabilities after randomizing each participant minimized imbalances in the number of accepting participants across arms over time. In the smoking cessation trial, unexpectedly large differences in acceptance among arms required us to limit the allocation of participants to less acceptable interventions. Nonetheless, the procedure achieved equal numbers of accepting participants in the more acceptable arms, and balanced the characteristics of participants across assigned interventions.

**Conclusions:**

Preference-adaptive randomization, coupled with analysis methods based on instrumental variables, can enhance the validity and generalizability of comparative effectiveness studies. In particular, preference-adaptive randomization augments statistical power by maintaining balanced sample sizes in efficacy analyses, while retaining the ability of randomization to balance covariates across arms in effectiveness analyses.

**Trial registration:**

ClinicalTrials.gov, NCT01526265; https://clinicaltrials.gov/ct2/show/NCT01526265 31 January 2012

## Background

Health-care providers, employers and insurers want to promote healthy behaviors, including medication adherence, tobacco cessation, weight loss and exercise [1-5]. The comparative effectiveness of any behavioral intervention depends on both its comparative acceptance (i.e., the probability that people assigned to an intervention will use it) and its comparative efficacy (i.e., how well the intervention works among people who use it) [6,7]:

(1) $${} \text{Effectiveness} :\,= \left[\text{Efficacy} \mid \text{Acceptance}\right] \times \text{Acceptance}.   $$

Therefore, it is essential to disentangle acceptance and efficacy to determine whether an intervention’s effectiveness is limited by low acceptance or low efficacy [8]. Unfortunately, disentangling acceptance and efficacy within randomized controlled trials is challenging. Simple randomization (or complete randomization) of participants to different interventions ensures that measures of effectiveness are not confounded by differences in the characteristics of individuals to whom the interventions are offered [9]. However, random assignment to interventions that yield different levels of acceptance can induce post-randomization losses that introduce bias and limit statistical power in standard analyses of the interventions’ comparative efficacy [10].

We recently designed a randomized controlled trial to compare the effectiveness of different financial incentive structures on prolonged smoking cessation (clinicaltrials.gov identifier: NCT01526265). Some of the financial incentive structures required participants to put some of their own money at risk, which might naturally be less attractive to participants than incentives with only upside potential. To promote the goals of achieving equal numbers of accepting participants in each arm and balancing the characteristics of participants across assigned interventions, we adapted the allocation probabilities throughout the enrollment period based on the inverse of the relative acceptance rates among randomized participants in each arm. Thus, our approach increased the likelihood that a participant was randomized to an intervention that had been rejected by previously randomized participants. Our preference-adaptive randomization procedure was intended to augment the statistical power of an instrumental variable analysis of efficacy [11], while reducing the potential for participant characteristics to bias an intention-to-treat analysis of effectiveness.

A large body of statistical literature has focused on adaptive designs for randomized trials. Brown and colleagues [12] conceptualized three types of adaptation: adaptive sequencing, which refers to the design of a new trial; adaptive designs, which refers to the conduct of an ongoing trial; and adaptive interventions, which refer to intervention experience of a study participant. Adaptive designs include covariate-adaptive randomization, in which allocation probabilities vary to minimize covariate imbalances across arms, and response-adaptive randomization, which uses the success or failure results on previously randomized participants in each arm to modify the allocation probabilities [13-15]. For example, in a randomized play-the-winner design, a participant is more likely to be randomized to an intervention deemed more successful based on the outcomes observed for previously randomized participants [16,17].

Our preference-adaptive randomization procedure shares elements of covariate- and response-adaptive randomization [18], but is distinct from each. On one hand, our randomization procedure could be viewed as response-adaptive. The acceptance analysis compares the relative proportions of participants who accept their assigned intervention. Acceptance among previously randomized participants is the response upon which the allocation probabilities are updated. On the other hand, our procedure could be viewed as a unique case of covariate-adaptive randomization. The efficacy analysis compares the relative rates of sustained smoking cessation among those who accept their assigned intervention. In the efficacy analysis, acceptance is conditioned as a special type of covariate. The special role of acceptance as both an effect and a cause in a comparative effectiveness study is similar to the role of the amount of treatment taken in a randomized encouragement design [19,20].

In this paper, we introduce a novel adaptive randomization procedure in which the allocation probabilities are updated based on the relative acceptance rates among randomized participants. Our goal is to elucidate the statistical and practical properties of adaptive randomization procedures, using comparative effectiveness studies as a motivating framework. In simulation studies, we determine the optimal frequency with which to update the allocation probabilities based on the number of participants randomized. We illustrate the development and application of our preference-adaptive randomization procedure, and demonstrate the benefits and challenges of an adaptive design, using the smoking cessation trial introduced above. We discuss instrumental variable methods that can be used to analyze the resultant efficacy data.

## Methods

We develop an adaptive randomization procedure in which the allocation probabilities are updated based on the inverse of the relative acceptance rates among randomized participants in each arm. In this approach, a participant is more likely to be randomized to an intervention that was deemed less acceptable among previously randomized participants. We accommodate stratified randomization by updating the allocation probabilities within each stratum, with the constraint that the sum of the probabilities within each stratum is 1.

Let *π*_*ijk*_ denote the allocation probability for arm (or intervention) *j*=1,…,*J* within stratum *i*=1,…,*I* at update *k*=1,…,*K*_*i*_. The total number of updates *K*_*i*_ could vary across strata due to differences in sample sizes. Let *n*_*ijk*_ denote the number of participants in stratum *i* who have accepted intervention *j* up to update *k*, with $n_{i\cdot k} = \sum _{j} n_{\textit {ijk}}$. The allocation probability for arm *j* within stratum *i* at update *k* can be calculated based on the accrued relative acceptance rates:

(2) $$ \pi_{ijk} = \pi_{ijk-1} \times \frac{n_{i\cdot k}}{n_{ijk}} \times \frac{1}{s_{i\cdot k}},  $$

for which $s_{i\cdot k} = n_{i\cdot k} \times \sum _{j^{\prime }} \pi _{ij^{\prime }k-1}/n_{ij^{\prime }k}$ denotes a scaling factor to ensure that $\sum _{j} \pi _{\textit {ijk}} =1$. Note that Equation (2) reduces to:

(3) $$ \pi_{ijk} = \pi_{ijk-1} \times \frac{1}{n_{ijk}} \times \frac{1}{\sum_{j^{\prime}} \pi_{ij^{\prime}k-1}/n_{ij^{\prime}k}}.  $$

Anticipated acceptance rates can be used to select the initial allocation probabilities *π*_*ij*0_, allowing for anticipated differences in acceptance within and across strata. In our smoking cessation trial, we assumed that the initial probabilities were constant across strata, but varied across arms within a stratum: *π*_1*j*0_=*π*_2*j*0_=⋯=*π*_*Ij*0_ ∀ *j*.

To implement our procedure, one must choose the frequency with which to update the allocation probabilities. Less frequent updating might not be responsive to differential acceptance rates across the interventions. More frequent updating might overcompensate for chance imbalances. Furthermore, it could require real-time data collection and analysis, which might not be feasible. In the following section, we use simulated data to determine the optimal frequency with which to update the allocation probabilities.

## Simulation studies

We performed simulation studies to determine the optimal frequency with which to update the allocation probabilities based on the number of participants randomized. The goal was to identify the updating interval that minimized imbalances in the numbers of accepting participants across arms over time. We hypothesized that insufficiently frequent updating would enable certain arms to grow disproportionately before change was enforced. Conversely, we hypothesized that overly frequent updating would be inefficient because, particularly early in the study, the procedure would overreact to variable acceptance rates, for which the denominators represented small samples of participants.

### Parameters

We designed the simulation studies to emulate our smoking cessation trial: a five-arm trial with a target sample size of 2,185 accepting participants. For simplicity, we did not assume stratified randomization. We considered updating intervals of 1, 3, 5, 10 and 20 participants. For example, with an updating interval of 3 participants, the allocation probabilities were updated after 3 participants were randomized since the last update. We defined the efficiency of an updating interval based on the sum of the squared deviations from an allocation probability of 0.2:

(4) $$ e(l) = \left[\,\sum\limits_{n=n_{0}}^{N} \sum\limits_{j=1}^{5} \left\{p_{jn}^{(l)}-0.2\right\}^{2}\right]^{-1},  $$

for which $p_{\textit {jn}}^{(l)}$ denotes the proportion of participants who have accepted intervention *j* among all participants who have accepted their assigned intervention at the time at which a total of *n*=*n*_0_,…,*N* participants have accepted their assigned intervention, under a randomization procedure with an updating interval of *l* participants. We selected *n*_0_=100 as a burn-in time, so that the efficiency was not influenced by early time periods during which the proportions in each arm were unstable. We selected *N* = 2,185 as the total number of participants who accept their assigned intervention. The efficiency measure in Equation (4) quantified the average imbalance in acceptance across arms over time, and is analogous to the efficiency measure proposed by Chen [21] for assessing sequential randomization schemes designed to balance the number of participants across arms. We defined the relative efficiency as the efficiency of an updating interval of *l* participants relative to that of an updating interval of 20 participants, i.e., *e*(*l*)/*e*(20), *l*=1,3,5,10. A relative efficiency >1 indicated increased efficiency.

We compared the efficiency of different updating intervals across a range of scenarios, in which we varied three primary factors:

1. *Acceptance probability for the most accepted intervention*: The true acceptance probability for the arm with the largest acceptance probability was 0.2, 0.4, 0.5, 0.6 or 0.8.
2. *Acceptance probabilities for less accepted interventions*: The acceptance probabilities for the arms other than the arm with the largest acceptance probability were divided evenly or unevenly. If divided evenly, then they were all set to 0.5 times the acceptance probability for the arm with the largest acceptance probability. If divided unevenly, then they were set to 0.2, 0.4, 0.6 and 0.8 times the acceptance probability for the arm with the largest acceptance probability.
3. *Initial values for acceptance probabilities*: The initial values for the acceptance probabilities:
  - *Correct*: Were equal to the true acceptance probabilities.
  - *More extreme*: Were unequal to the true acceptance probabilities, with the initial value for the arm with the largest acceptance probability set to 1.2 times its true value and the remaining initial values set to 0.8 times their true values.
  - *Less extreme*: Were unequal to the true acceptance probabilities, with the initial value for the arm with the largest acceptance probability set to 0.8 times its true value and the remaining initial values set to 1.2 times their true values.
  The initial allocation probabilities were proportional to the inverse of the initial acceptance probabilities.

We performed 500 iterations for each of the 30 scenarios in the full 5×2×3 factorial design. Both overall and for each of the three primary factors, we summarized the results by calculating an average relative efficiency (ARE); for each scenario, we calculated the ARE for that scenario and then averaged the AREs across scenarios. Simulations were performed using R 3.1.0 (R Development Core Team, Vienna, Austria), including the e1071 extension package.

### Results

Across all 30 scenarios in the full 5×2×3 factorial design, the average ARE for an updating interval of 1, 3, 5 and 10 participants (compared to an updating interval of 20 participants) was 1.21, 1.11, 1.08 and 1.03, respectively, which indicated that, on average, more frequent updating led to increased efficiency. On average, an updating interval of 1 participant was 21% more efficient than an updating interval of 20 participants. Table 1 provides the average ARE according to the acceptance probability for the most accepted intervention. Note that a lower acceptance probability for the most accepted intervention corresponded to lower acceptance probabilities among all interventions. The efficiency gain of more frequent updating was lower for lower values of the acceptance probabilities. If acceptance was lower, then more frequent updating was more sensitive to individual responses and therefore led to larger imbalances in the number of accepting participants across arms over time.

**Table 1:** **Average efficiency relative to an updating interval of 20 participants according to the acceptance probability for the most accepted intervention**

**Updating interval**	**Acceptance probability for most accepted intervention**				
	**0.2**	**0.4**	**0.5**	**0.6**	**0.8**
1	1.14	1.19	1.21	1.26	1.28
3	1.09	1.10	1.11	1.12	1.12
5	1.05	1.06	1.06	1.08	1.08
10	1.02	1.02	1.03	1.03	1.02

Table 2 provides the average ARE according to the distribution of the acceptance probabilities for the less accepted interventions. The efficiency gain of more frequent updating was higher for an uneven distribution, which indicated that more frequent updating was more efficient when there were more substantial differences among acceptance probabilities. Table 3 provides the average ARE according to the initial values for the acceptance probabilities. There were no substantial differences in the average ARE when the initial values were correct or incorrect, which indicated that the randomization procedure recovered from incorrect initial values.

**Table 2:** **Average efficiency relative to an updating interval of 20 participants according to the distribution of the acceptance probabilities for the less accepted interventions**

**Updating interval**	**Acceptance probabilities for**	
	**less accepted interventions**	
	**Even**	**Uneven**
1	1.16	1.26
3	1.08	1.15
5	1.05	1.11
10	1.02	1.05

Even: All set to 0.5 times the acceptance probability for the arm with the largest acceptance probability. Uneven: Set to 0.2, 0.4, 0.6 and 0.8 times the acceptance probability for the arm with the largest acceptance probability.

**Table 3:** **Average efficiency relative to an updating interval of 20 participants according to the initial values for the acceptance probabilities**

**Updating interval**	**Initial values for acceptance probabilities**		
	**Correct**	**More extreme**	**Less extreme**
1	1.20	1.20	1.21
3	1.11	1.11	1.11
5	1.09	1.07	1.08
10	1.03	1.04	1.03

Correct: Equal to the true acceptance probabilities. More extreme: Unequal to the true acceptance probabilities, with the initial value for the arm with the largest acceptance probability set to 1.2 times its true value and the remaining initial values set to 0.8 times their true values. Less extreme: Unequal to the true acceptance probabilities, with the initial value for the arm with the largest acceptance probability set to 0.8 times its true value and the remaining initial values set to 1.2 times their true values.

Results were similar (within 1% to 2%) when no burn-in time was used to calculate the efficiencies.

### Summary

Our preference-adaptive randomization procedure – based on the inverse of the relative acceptance rates among randomized participants in each arm – performed best with frequent updating, accommodated differences in acceptance across interventions, and was robust to incorrect initial values. An updating interval of 1 participant performed well across all scenarios considered.

## Illustration

### Background

We designed a randomized controlled trial of smoking cessation interventions among CVS/Caremark employees and their friends and family members. The goal of the study was to compare usual care (e.g., access to online information on smoking cessation, access to phone-based cessation assistance and nicotine replacement therapy) with usual care plus one of four financial incentive structures likely to vary in their appeal to participants. Participants in the individual reward arm received a financial reward if they achieved sustained abstinence. In the collaborative reward arm, a participant was assigned to a group of six participants; a participant’s financial reward for sustained abstinence was increased as the abstinence rate among group members increased. Participants in the individual deposit arm deposited money at the beginning of the study; if they achieved sustained abstinence, then they received their deposit plus a financial reward. In the competitive deposit arm, a participant was assigned to a group of six participants; deposits were redistributed among only those group members who achieved sustained abstinence.

### Methods

The target sample size was 2,185 smokers (437 per arm) who would accept their assigned intervention. Over a 9-month enrollment period, potential participants were recruited via a web-based research portal [22]. Participants were told that the smoking cessation interventions involved the use of financial incentives, but specific details of the interventions were not provided. Once randomized, participants received a detailed description of their assigned intervention. We defined the acceptance rate as the proportion of participants randomized to each of the four incentive arms who, after learning the details of the incentive structure to which they were assigned, agreed to the contract. In the usual care arm, acceptance was assumed for everyone because no new intervention was offered. Participants who accepted the collaborative reward or competitive deposit intervention were subsequently assigned to a group of six participants. Because group assignment occurred after acceptance, it was not necessary to adjust for group effects when calculating acceptance rates. All participants provided informed consent. The University of Pennsylvania Institutional Review Board approved the study protocol.

We stratified the randomization by two dichotomous variables: whether or not participants had full health-care benefits through CVS/Caremark; and annual household income above or below $60,000. Initial allocation probabilities across strata were: usual care, 0.20; individual reward, 0.15; collaborative reward, 0.15; individual deposit, 0.25; and competitive deposit, 0.25. This unbalanced randomization was intended to account for hypothesized differences in acceptance rates across arms; in particular, we predicted that participants would be less likely to accept deposit contracts because those require participants to put up some of their own money at the start, and risk not getting it back if they do not succeed in quitting. To further promote the goals of achieving equal numbers of accepting participants in each arm and balancing the characteristics of participants across assigned interventions, we also adapted the allocation probabilities within strata throughout the enrollment period. Although our simulation studies indicated that an updating interval of 1 participant performed well, we used an updating interval of 3 participants to reduce the complexity of the required programming in the web-based portal.

### Results

**Figure 1:**
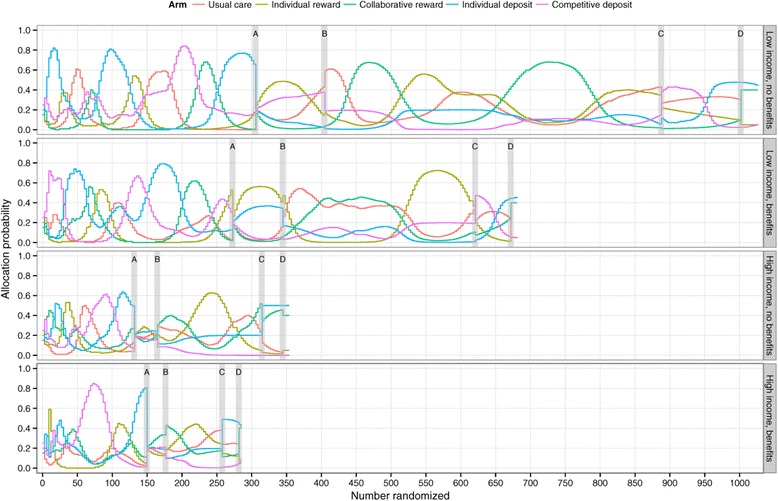
**Probability of being allocated to each incentive structure in each of the four strata over the enrollment period.** A, B, C, and D indicate protocol changes in the randomization procedure. These changes were made to address observed disparities in acceptance rates. On 11 April 2012, we implemented a 60%/40% split between [usual care + individual reward + collaborative reward arms] and [individual deposit + competitive deposit arms] (A). On 25 April 2012, we changed to an 80%/20% split between the same two groups (B). On 14 September 2012, we changed to a 50%/50% split between the same two groups (C). On 26 September 2012, we retained this 50%/50% split, continued the adaptive randomization for the deposit arms, but among the 50% of participants randomized to the group containing the other three arms, we fixed the allocation probabilities for the usual care (15%), individual reward (15%) and collaborative reward (70%) arms to bolster assignment to the latter (D).

The preference-adaptive randomization procedure we implemented resulted in marked variations in the proportions of participants allocated to the different study arms over time (Figure 1). For example, in the most populous of the four strata (i.e., participants with annual household incomes less than $60,000 who also lacked CVS/Caremark benefits, or low income, no benefits), the percentage of participants allocated to the individual deposit arm varied from <5% to >80% during the initial enrollment period. During this same time, allocation probabilities for each of the arms exceeded 50% at one or more points in time, and each dropped to <15% at other times.

Following the first 7 weeks of recruitment, with nearly 1,000 participants enrolled, we recognized that the interventions had differences in acceptance rates much larger than we originally hypothesized. We concluded that if we did not modify the randomization procedure, so many participants would be diverted to the least acceptable interventions that we would fail to adequately enroll any of the arms because of limitations on eligible participants. To combat this problem, we implemented a series of restrictions on the allocation probabilities. We set ceilings for the proportion of participants allocated to the least popular arms (i.e., individual deposit and competitive deposit). After this initial change 7 weeks into the study (denoted by A in Figure 1), we made subsequent modifications 9, 29 and 31 weeks into the study (denoted by B, C and D, respectively, in Figure 1).

Our preference-adaptive randomization procedure produced balance in the numbers of accepting participants in the three arms that were reasonably well accepted by participants (i.e., usual care, individual reward and collaborative reward). Specifically, we achieved the target sample size of at least 437 participants in each of those arms, with negligible differences in the numbers of accepting participants among those arms. The procedure also successfully balanced the characteristics of participants across assigned interventions. Indeed, of the more than 30 participant characteristics that we measured, none revealed an important imbalance across arms. Only one variable, ethnicity, was statistically unbalanced (*P*=0.042).

Balance across arms was not uniformly achieved when evaluating only participants who accepted their assigned intervention (Table 4). In particular, annual household income was highly imbalanced, with an over-representation of high-income individuals in the individual deposit and competitive deposit arms (*P* <0.001). These results provide support for the concern that analyses based on participants who accepted their assigned intervention would be susceptible to selection effects. In the following section, we discuss instrumental variable methods that can address such selection effects.

**Table 4:** **Participant characteristics by accepted intervention**

	**Usual**	**Individual**	**Collaborative**	**Individual**	**Competitive**	***P***
	**care**	**reward**	**reward**	**deposit**	**deposit**	
	***n*** **=468**	***n*** **=472**	***n*** **=442**	***n*** **=75**	***n*** **=71**	
Age, years	34 (26, 47)	33 (25, 46)	32 (25, 45)	41 (27, 51)	34 (26, 43)	0.032
Female sex, *n* (%)	300 (64)	301 (64)	278 (63)	38 (51)	42 (59)	0.23
Race, *n* (%)						0.090
Caucasian	365 (78)	389 (82)	328 (74)	62 (83)	60 (85)	
African American	43 (9)	42 (9)	55 (12)	5 (7)	4 (6)	
Other	60 (13)	41 (9)	59 (13)	8 (11)	7 (10)	
Hispanic ethnicity, *n* (%)	42 (9)	30 (6)	23 (5)	4 (5)	4 (6)	0.21
Annual household income, *n* (%)						<0.001
<$20,000	114 (24)	111 (24)	117 (26)	10 (13)	8 (11)	
$20,000 to $39,999	143 (31)	154 (33)	139 (31)	16 (21)	16 (23)	
$40,000 to $59,999	89 (19)	93 (20)	71 (16)	11 (15)	13 (18)	
$60,000 to $79,999	59 (13)	49 (10)	41 (9)	13 (17)	11 (15)	
$80,000 to $99,999	28 (6)	25 (5)	34 (8)	8 (11)	6 (8)	
≥$100,000	35 (7)	40 (8)	40 (9)	17 (23)	17 (24)	
CVS/Caremark benefits, *n* (%)	188 (40)	199 (42)	176 (40)	41 (55)	37 (51)	0.051
Years as a regular smoker	15 (6, 26)	12 (6, 25)	13 (6, 25)	17 (6, 30)	15 (7, 24)	0.49
Cigarettes smoked per day	15 (10, 20)	15 (10, 20)	15 (9, 20)	15 (10, 20)	15 (10, 20)	0.62

Summary statistics are median (25th, 75th percentile) unless otherwise noted as *n* (%).

### Summary

We designed a randomized controlled trial to compare the effectiveness of different financial incentive structures on prolonged smoking cessation. We implemented a preference-adaptive randomization procedure in which allocation probabilities within strata were updated after every third participant based on the relative acceptance rates among randomized participants. The procedure required direct modification during the enrollment period, but nevertheless achieved its stated goals. First, for the three arms we targeted for complete enrollment, we achieved equal numbers of accepting participants in each arm, which will enhance the power of the efficacy analyses. Second, we achieved balance in the characteristics of participants across assigned interventions, which will reduce the potential for bias in the effectiveness analysis.

## Estimation of treatment effects

In previous sections, we described the development and implementation of a preference-adaptive randomization procedure in comparative effectiveness studies, using a smoking cessation trial as an illustrative example. In this section, we outline an analysis plan to estimate acceptance, efficacy and effectiveness; see Equation (1).

A standard intention-to-treat analysis is used to compare the treatments’ acceptance and effectiveness. For evaluating efficacy, a standard per-protocol analysis would compare the cessation rate among participants who accept intervention *j* to those who receive the control (with or without inclusion of those who were assigned intervention *j* but declined it). However, the standard per-protocol analysis could be subject to selection bias if smokers who do not accept an incentive differ from those who do in ways that relate to their probabilities of quitting [23]. To address such selection effects, we model the randomization arm as an instrumental variable [11]. In the instrumental variable approach, the cessation rate of each intervention is adjusted for the percentage of participants who accept their assigned intervention, thereby estimating complier-averaged causal effects and attenuating the selection effects [24,25].

A key advantage of the instrumental variable approach over a standard per-protocol analysis is that the instrumental variable approach uses the data on all randomized participants, rather than merely those who accept their assigned intervention. Therefore, the instrumental variable approach adheres to the randomized trial principle that participants should be analyzed according to their randomization status, rather than according to their self-selected acceptance status. For the instrumental variable analysis, we will use a two-stage least squares linear probability model [26]. By using the preference-adaptive randomization procedure, which balances the number of accepting participants in each arm (or, in our application, the arms targeted for complete enrollment), we increase the power for the instrumental variable analysis.

## Discussion

In this paper, we introduced a preference-adaptive randomization procedure in which the allocation probabilities were updated based on the inverse of the relative acceptance rates among randomized participants in each arm. We showed that the procedure performed best with frequent updating, accommodated differences in acceptance across interventions, and was robust to incorrect initial values. We applied our procedure to a randomized controlled trial to compare the effectiveness of different financial incentive structures on prolonged smoking cessation. The randomization procedure strengthened the trial in at least three ways. First, the procedure highlighted the very low acceptance rates in certain arms because it resulted in correspondingly high allocation probabilities to those less acceptable arms. This enabled us to modify the randomization procedure during enrollment to preserve the possibility of fully enrolling more acceptable arms. Second, despite producing large variations in the allocation probabilities within and across arms over time, the procedure yielded comparable numbers of accepting participants across the three arms that we allowed to enroll appreciable numbers of participants, as well as across the two arms in which allocation was restricted when they were found to be less acceptable. This across-arm balance will maximize statistical power. Third, the procedure achieved balance in the observed characteristics of participants across assigned interventions, which increases confidence that unmeasured characteristics (e.g., motivation to quit) would also be balanced in the effectiveness analysis. Balance across arms was not uniformly achieved when evaluating only participants who accepted their assigned intervention, which provides support for the concern that such analyses would be susceptible to selection effects. We discussed instrumental variable methods that can address such selection effects.

Although our preference-adaptive randomization procedure performed well in simulation studies and in our application, there are limitations to the procedure’s application. First, our simulation studies indicated that an updating interval of 1 participant was optimal. In practice, however, such frequent updating could require a sophisticated data-transmission and storage infrastructure to perform rapid data collection and analysis. In our smoking cessation trial, we programmed the procedure into a web-based portal that was used for data collection and randomization. Second, in our application the procedure required manual modification due to lower-than-anticipated acceptance of less appealing arms, and correspondingly higher-than-anticipated automatic adjustments to the allocation probabilities for those arms. Left unchecked, those automatic adjustments would have hampered our ability to adequately enroll any of the arms. Future investigators might wish to program automatic modifications in their preference-adaptive randomization procedure, similar to the modifications that we made manually (e.g., ceilings for the allocation probabilities). Third, our procedure could introduce confounding because allocation probabilities might depend on a complicated function of time (within strata). If the response also varies over time, then differences in the average response between arms could be confounded by temporal trends. Therefore, when using this approach in practice, it might be prudent to adjust for temporal trends (within strata) using a flexible specification for calendar time, such as regression splines. Fourth, like many adaptive designs or interim analyses, preference-adaptive randomization might require that a member of the study team, such as a statistician, be unblinded during the trial. Unblinding requires careful consideration of the statistician’s role in the study’s conduct and reporting.

Our adaptive design, coupled with appropriate statistical analysis methods, could be used to enhance the validity and generalizability of any comparative effectiveness study, blinded or unblinded, in which study participants choose to adhere to their assigned intervention [9,27]. Examples include large simple trials of vaccines or virtually any pharmaceutical for which adherence might not be 100%, and, of course, trials of almost any behavioral intervention. However, application of our procedure requires consideration of two key features of adherence: the time lag between randomization and measurement of adherence; and whether adherence is assessed as a dichotomous variable, or as an adherence proportion or rate. In our smoking cessation trial, acceptance was measured immediately after randomization as being present or absent. By contrast, in drug trials, adherence might not be measured until several weeks or months after randomization, and might be measured as a proportion of pills taken among those prescribed.

Future research is needed to determine how a time lag in the assessment of adherence influences the efficiency of preference-adaptive randomization and the optimal updating interval. Conceivably, with more distant measurements of adherence, more frequent updating would be even more advantageous so that allocation probabilities can be modified as soon as adherence data become available. Research is also needed to determine how best to handle situations in which adherence is measured as a proportion. The easiest, but perhaps least precise, approach would be to set an adherence threshold, thereby converting adherence to a dichotomous variable. However, more complex approaches, in which progressively large differences in observed adherence rates result in progressively large feedback influences on the allocation probabilities, could also be developed. The potential applicability of such strategies is quite broad, but requires further testing to ensure that balance would still be achieved across randomized arms.

## Conclusions

In comparative effectiveness studies, frequent updating of allocation probabilities – based on the inverse of the relative acceptance rates among randomized participants in each arm – augments statistical power by maintaining balanced sample sizes in efficacy analyses, while retaining the ability of randomization to balance covariates across arms in effectiveness analyses. Preference-adaptive randomization, coupled with statistical analysis methods based on instrumental variables, could be used to enhance the validity and generalizability of any comparative effectiveness study in which study participants choose to adhere to their assigned intervention.

## Abbreviation

ARE: Average relative efficiency
